# Exhaled Volatile Organic Compounds in Ischemic Cardiomyocytes: Signatures of Oxidative Stress!

**DOI:** 10.31083/RCM49046

**Published:** 2026-04-24

**Authors:** Basheer Abdullah Marzoog, Philipp Kopylov

**Affiliations:** ^1^Institute of Personalized Cardiology of The Center “Digital Biodesign and Personalized Healthcare” of Biomedical Science and Technology Park, Sechenov First Moscow State Medical University, 119991 Moscow, Russia

**Keywords:** ischemic heart disease, oxidative stress, myocardial infarction, volatile organic compound, homeostasis, metabolome, inflammasome

## Abstract

Volatile organic compounds (VOCs) reflect the homeostatic state of an organism, including that of cardiomyocytes. Ongoing alterations in cardiac muscle tissue can be detected as VOCs in exhaled breath. Traditionally, aging and the presence of atherosclerosis in the coronary arteries are associated with the development of ischemic heart disease (IHD). Pathophysiologically, IHD is characterized by an imbalance between the demand for and supply of oxygen and nutrients to cardiomyocytes. This imbalance favors oxidative stress over the antioxidant defense system, leading to the accumulation of reactive oxygen species (ROS) in cardiomyocytes. The molecular mechanisms of IHD involve the peroxidation of lipids, proteins, and nucleotides in cellular and intercellular components, particularly mitochondrial membranes. The products of peroxidation are released into the circulation and eventually reach the pulmonary circulation, where these products are exhaled as VOCs. This suggests that changes in exhaled VOCs in patients with IHD likely arise from oxidative stress within cardiomyocytes.

## 1. Introduction

Ischemic heart disease (IHD) is a major global challenge requiring solutions for early detection, prevention, and risk reduction. Current censuses have demonstrated a predominance of IHD as a cause of mortality and morbidity among other causes of cardiovascular diseases [1, 2]. Hence, developing new strategies to combat the ongoing rise in IHD incidence worldwide is critically important. Several methods have been developed for early prediction of IHD risk in the general population; among these, risk scores such as SCORE, SCORE2, SCORE 2-OP, and the SMART risk score are used for individuals with previously diagnosed IHD [3, 4, 5]. Furthermore, new tools have been developed to assess the prognosis and risk of various cardiometabolic diseases, including IHD and left ventricular diastolic dysfunction [6, 7]. One such tool is the single-channel electrocardiogram (Cardio-Qvark) [8, 9]. Another emerging approach for studying IHD includes exhaled breath analysis, which examines biochemical components, specifically volatile organic compounds (VOCs) [10].

Exhaled VOCs are organic compounds released during expiration that reflect various metabolic processes within the body [7, 10, 11]. These compounds can originate from endogenous sources, such as metabolic byproducts of cellular processes, or from exogenous sources, including environmental exposure and dietary intake [12, 13]. Exhaled breath is highly complex, containing hundreds of different VOCs that can provide insight into the health status of an individual [14]. Thus, exhaled VOCs can serve as a mirror of cardiac homeostasis and, in particular, of cardiomyocyte health at the subcellular and cellular levels [8]. The use of exhaled VOCs in medical practice remains underappreciated and requires further work toward unification and the development of a single universal guideline (standardization). Indeed, research has shown that specific exhaled VOC profiles can be associated with diseases such as cancer, respiratory diseases, and metabolic disorders [13]. The heterogeneity of exhaled VOCs is due to variations in methods and techniques, as well as in devices and protocols [15]. To address these issues, universal clinical recommendations must be developed. The production of VOCs is closely related to biochemical processes in the body [14, 16]. For example, oxidative stress and inflammation can alter VOC profiles, making them potential biomarkers for various diseases. Understanding these mechanisms is crucial for accurately interpreting VOC data [17].

Oxidative stress plays a crucial role in the production of VOCs in cardiomyocytes during IHD [18, 19, 20, 21]. Reactive oxygen species (ROS) generated under oxidative stress can induce lipid peroxidation, protein damage, and DNA damage, leading to the release of specific VOCs into exhaled breath [18, 19, 20]. Several studies have identified VOCs associated with oxidative stress in patients with IHD [18, 19, 20]. Moreover, oxidative stress in cardiomyocytes is a key contributor to VOC production during IHD, and the interaction between oxidative damage and VOC generation is crucial in the pathogenesis of the disease [22]. Targeting oxidative stress, alongside exhaled VOC analysis, holds promise for developing more effective therapeutic strategies and improving the management of IHD in individuals at elevated risk [22].

Despite advancements in breath analysis technology, several gaps remain, including variability in results, lack of standardization, and limited clinical validation [23, 24, 25]. The rationale for utilizing exhaled VOCs as biomarkers lies in the associated noninvasive nature and ability to provide real-time insights into metabolic changes. Indeed, unlike traditional diagnostic methods that may require invasive procedures or complex sample processing, breath analysis offers a simpler and faster alternative. This makes this analysis particularly attractive for monitoring chronic conditions and evaluating treatment responses.

## 2. Oxidative Stress in Cardiac Homeostasis 

Oxidative stress plays a crucial role in the development and progression of cardiac diseases, including hypertrophy, IHD, and heart failure [26, 27, 28, 29, 30, 31]. ROS and reactive nitrogen species (RNS) are highly reactive molecules that can cause cellular and subcellular abnormalities when RNS production exceeds the buffering capacity of antioxidant defense systems [26, 28].

In the heart, oxidative stress activates pro-hypertrophic and remodeling signaling cascades, leading to cardiac hypertrophy [29]. Oxidative stress also triggers protein damage, the formation of toxic protein oligomers, protein aggregation, and the accumulation of oxidized proteins, which can alter cardiac function [28]. Moreover, oxidative stress can lead to lipid peroxidation, DNA damage, and oxidation of proteins and other macromolecules, culminating in pathological myocardial remodeling, fibrosis, and contractile dysfunction [28].

Dysregulation of autophagy, the process responsible for removing damaged cellular components, has also been implicated in the interaction between oxidative stress and cardiovascular disease [28, 32, 33, 34]. Careful regulation of ROS/RNS levels and subsequent interaction with autophagic degradation pathways is crucial for maintaining cardiac homeostasis [28].

Several sources of oxidative stress have been identified in the heart, including *NADPH* oxidases (*Nox*), uncoupled nitric oxide synthases, xanthine oxidase, cyclooxygenases, and cytochrome P450 enzymes, as well as mitochondrial sources such as the respiratory chain, monoamine oxidases (MAOs), *p66shc*, and *NOX4* [26, 29]. Targeting these sources, as well as improving antioxidant defenses, has been explored as a potential therapeutic strategy for preventing and treating cardiac diseases [29, 30].

ROS, including superoxide anion, hydroxyl radical, and hydrogen peroxide, are critical signaling molecules in the heart. ROS regulate essential functions, including heart development, cardiomyocyte maturation, and vascular tone, under normal conditions. However, excessive ROS production can lead to oxidative stress, which damages DNA, proteins, and lipids, and contributes to various cardiac diseases, such as ischemia-reperfusion injury [35]. One of the critical intracellular organelles involved in regulating oxidative stress is the mitochondrion. Mitochondria are central to energy production and are a significant source of ROS. The mitochondrial respiratory chain and other mitochondria-localized proteins contribute to the ROS pool. To manage oxidative stress, mitochondria have a network of ROS-scavenging systems that mitigate this stress, highlighting the importance of mitochondrial function in cardiac health [35].

Oxidative stress is a key mechanism underlying the development and progression of IHD; thus, modulating oxidative stress holds promise for preventive and therapeutic interventions aimed at cardiovascular homeostasis [36, 37, 38, 39].

## 3. VOCS Change in Patients With IHD

Patients with IHD undergo metabolic changes as an adaptation to reduced nutrient supply and as a consequence of ischemic injury [40, 41, 42]. Accordingly, these metabolic changes typically manifest as molecular biomarkers in the bloodstream. The molecular byproducts released by ischemic cardiomyocytes likely continue to circulate in the pulmonary circulation and are subsequently exhaled as VOCs.

The current issue facing the scientific community is developing guidelines for exhaled VOCs and identifying those known to date across different pathologies.

Recent findings have shown that patients with a positive myocardial perfusion defect on stress computed tomography myocardial perfusion imaging correlated with VOCs with the following mass/charge ratios: 94.0537307640158 (C_2_H_7_NO_3_H^+)^, 144.91780932607332, 87.93367781464133, 87.07684847044642 (2-pentanone or 3-methyl-2-butanone), 72.05367879285716 (C_4_H_8_O^+^) [43].

## 4. Role of Oxidative Stress in VOCs From Patients With IHD

Oxidative stress plays an important role in the production of VOCs in patients with IHD [16, 21, 44, 45]. ROS generated during oxidative stress can lead to lipid peroxidation, DNA damage, and protein oxidation, resulting in the release of specific VOCs [16, 44].

The release of these VOCs can be detected in the breath, urine, or blood of patients with IHD [18, 20, 21]. Therefore, monitoring changes in the levels of these VOCs can provide information on the severity of oxidative stress and can potentially serve as noninvasive biomarkers for IHD [16, 18, 19, 20, 21]. However, the effect of oxygen on oxidative stress markers in IHD remains unclear, as previous studies have yielded conflicting results [21, 46]. Thus, more research is needed to identify the specific VOCs associated with oxidative stress in IHD and to evaluate the potential of using VOCs as diagnostic and prognostic tools [18, 21].

Several studies have identified volatile organic compounds (VOCs) associated with oxidative stress in ischemic heart disease (IHD), including ethane, pentane, aldehydes, and acetonitrile [16, 18, 20, 21, 44]. Ethane and pentane are direct end-products of reactive oxygen species (ROS)-induced lipid peroxidation, reflecting oxidative degradation of polyunsaturated fatty acids [16, 20, 21]. In contrast, aldehydes such as benzaldehyde and dimethylbenzaldehyde are generated as secondary byproducts of lipid peroxidation [18, 44]. Acetonitrile has also been tentatively proposed as a marker of oxidative stress in patients with IHD [18, 44].

## 5. The Dilemma of Oxidative Stress–VOC Axis in the Pathogenesis of IHD

The relationship between exhaled VOCs and oxidative stress in the pathogenesis of IHD is complex and is not yet fully understood [16, 19, 30].

Oxidative stress plays a crucial role in the development and progression of IHD [19, 30, 31]. ROS can cause cellular and molecular abnormalities, leading to lipid peroxidation, protein damage, and DNA damage [19, 30]. This oxidative stress activates pro-hypertrophic and remodeling signaling cascades, contributing to cardiac hypertrophy and contractile dysfunction [30].

Specific VOCs have been associated with oxidative stress in patients with IHD [16, 19, 20]. Hence, monitoring changes in the levels of these VOCs can provide insight into the severity of oxidative stress and potentially serve as noninvasive biomarkers of IHD [19, 20, 46].

However, the effect of oxygen on oxidative stress markers in IHD remains unclear, as previous studies have yielded contradictory results [46]. Consequently, more research is needed to identify the specific VOCs associated with oxidative stress in IHD and to evaluate the potential of using these VOCs as diagnostic and prognostic tools [16, 19, 30].

Although oxidative stress is clearly linked to the pathogenesis of IHD, the precise mechanisms and the potential of VOCs as biomarkers remain to be elucidated, necessitating further research to resolve the current dilemma.

## 6. Current Perspective on the Role of Cardiomyocytes in Oxidative Stress in Patients With IHD 

The current understanding of oxidative stress in myocardial cells (cardiomyocytes) in patients with IHD is a topic of significant research and clinical interest. Oxidative stress, characterized by an imbalance between ROS and antioxidant defense mechanisms, plays a crucial role in the pathogenesis and progression of IHD [19, 31, 47, 48].

In patients with IHD, oxidative stress in cardiomyocytes can cause cellular damage, dysfunction, and apoptosis, contributing to the development of myocardial ischemia and subsequent complications [19, 48]. The intricate interaction between oxidative stress and cardiomyocytes involves the generation of ROS, lipid peroxidation, protein oxidation, and DNA damage, all of which can affect cell function and viability [19, 48].

Prior research has highlighted the importance of biomarkers related to oxidative stress in understanding the pathogenesis and predicting the clinical outcomes of IHD [31]. In particular, biomarkers such as reactive oxygen metabolites (ROMs) and total antioxidant capacity (OXY) have shown promise in quantifying oxidative stress levels and assessing the severity of myocardial damage in patients with IHD [31].

Therapeutically, targeting oxidative stress in cardiomyocytes may enable more effective treatment strategies for IHD [30]. However, a comprehensive understanding of the specific roles of ROS in the pathophysiological mechanisms of IHD, as well as the overall interactions with other signaling systems, is essential for the successful development of targeted therapeutic approaches with minimal adverse effects [31, 48].

Recent advancements in noninvasive diagnostic modalities have introduced the modified Haller index (MHI), which has been tested for its efficacy in identifying individuals at low risk of coronary artery disease (CAD) [49]. A recent study suggests that the MHI, traditionally used to assess chest wall deformities, may also serve as a predictive tool for CAD [49]. This suggests that using the chest transverse diameter rather than the distance between the sternum and spine may correlate with CAD, offering a novel perspective on the relationship between body structure and heart disease. Thus, the use of MHI could represent a significant step forward in the early screening and management of CAD, potentially reducing the need for more invasive diagnostic procedures.

The current perspective underscores the critical role of oxidative stress in cardiomyocytes in the context of IHD, emphasizing the need for further research to elucidate the specific mechanisms and interactions involved, ultimately paving the way for more effective therapeutic interventions for IHD.

## 7. Current Challenges and Future Therapeutic Strategies Using the VOC–Cardiomyocytes Oxidative Stress Axis 

The interaction between VOCs and oxidative stress in myocardial cells (cardiomyocytes) presents both challenges and opportunities for the prevention and early diagnosis of IHD [50].

Current challenges include establishing specific exhaled VOC biomarkers related to IHD. Although certain exhaled VOCs, such as ethane, pentane, and aldehydes, have been associated with oxidative stress in patients with IHD, further research is needed to identify specific and reliable biomarkers [16, 20, 21, 30]. Moreover, the precise mechanisms linking exhaled VOCs to oxidative stress in cardiomyocytes during the pathogenesis of IHD remain poorly understood, warranting additional investigations [16, 21, 30]. Additionally, resolving conflicting results, where previous studies have yielded conflicting results on the effect of oxygen on markers of oxidative stress in IHD, highlights the need for more consistent and robust data [16, 51].

The diagnosis of IHD using exhaled VOCs represents a cutting-edge approach that leverages metabolic changes in the body [10]. However, despite the innovative nature of this method, practical implementation faces several challenges, including the cost and sensitivity of the required instruments, as well as the complexity of integrating this technology into routine clinical workflows [52]. Although the potential of exhaled VOC-based diagnostics for IHD is significant, addressing these nontrivial challenges necessitates a concerted effort from researchers, clinicians, and policymakers [53]. Investment in research and development, training, and infrastructure is essential to overcome these barriers, and as technology matures and costs decrease, integrating VOC-based diagnostics into clinical practice may become more feasible, ultimately leading to improved patient outcomes in the management of IHD [54].

Future therapeutic strategies include targeting key sources of oxidative stress in cardiomyocytes, such as NADPH oxidases (Nox), mitochondria, and uncoupled nitric oxide synthases, which could lead to novel therapeutic interventions [30, 51]. Furthermore, improving the antioxidant defense system to enhance cardiomyocyte antioxidant capacity, through dietary supplementation or pharmacological interventions, can help mitigate the detrimental effects of oxidative stress [30, 51]. Moreover, developing a reliable, sensitive method to detect and monitor changes in exhaled VOC levels associated with oxidative stress could enable early diagnosis, risk stratification, and personalized treatment approaches for patients with IHD [16, 20, 21]. Additionally, combining exhaled VOCs analysis with other diagnostic tools and modalities, such as imaging techniques, single-lead electrocardiography, and biochemical assays, may provide a more comprehensive assessment of myocardial health and guide therapeutic decision-making [16, 21]. Although the oxidative stress axis in cardiomyocytes poses challenges in establishing specific biomarkers and elucidating precise mechanisms, this axis also offers promising avenues for future therapeutic strategies targeting oxidative stress and for using VOC analysis to improve the diagnosis and treatment of IHD.

## 8. Discussion 

Classically, IHD develops against a background of stable atherosclerosis in CAD [55, 56]. Typically, these patients have existing risk factors for IHD, such as obesity [16, 21, 57]. Moreover, obesity is well known to be associated with mild chronic inflammation and the release of ROS due to lipid peroxidation; thus, obese individuals with primary central obesity are well known to exhibit markedly elevated oxidative stress, accompanied by increased levels of isoprostanes (F2-IsoP), F2-8-isoprostaglandin F2α (8-isoPGF2 α), malondialdehyde (MDA), and protein carbonylation, a biomarker reflecting the degree of fat storage [58]. Although people with a tall, slender build may generally have lower levels of oxidative stress than those with obesity, many other factors, such as lifestyle choices, genetic predisposition, metabolic rates, and individual health conditions, also play important roles when discussing oxidative stress across different body types [59]. However, in theory, changes in the components of exhaled VOCs should be identical, as these molecules originate from ischemic cardiomyocytes. However, in practice, factors including obesity, genetic predisposition, gut microbiota, metabolic rate, and other health conditions can slightly alter the components of exhaled breath analysis in patients with IHD.

Therefore, the metabolic byproducts of oxidative stress are not necessarily derived from ischemic cardiomyocytes [60]. To address the current problem, it is suggested to conduct an additional study in patients with metabolic syndrome and in other groups without metabolic syndrome, and to determine the associated exhaled VOCs in individuals with metabolic syndrome.

A single study in patients with potential acute coronary syndrome demonstrated that breathome analysis can be used as a noninvasive tool to discriminate patients with CAD [61]. However, the study lacked clearly defined inclusion and exclusion criteria, which introduced potential bias and reduced the overall rigor of the work.

Some findings suggest that basal pentane levels are less useful than lipid peroxide levels (malondialdehyde; MDA) as an index of lipid peroxidation in patients with CAD, and that breath pentane is a sensitive index of reperfusion-induced lipid peroxidation [62].

The current challenge for scientists is to determine whether the detected VOCs are due to IHD (cardiomyocyte ischemia), lipid peroxidation, atherosclerotic plaque, or simply to changes in the gut microbiota. Therefore, this question remains open, and no clinical studies provide clear answers. Our previous study identified some important VOCs but did not clarify the pathophysiological pathways of these VOCs. However, we suggest that VOCs originate from the ischemic heart rather than from other parts of the organism.

Several limitations reduce the viability of exhaled breath analysis for the diagnosis of IHD, including the lack of standardization of protocols for sample collection and analysis, sampling conditions, storage, and analytical platforms, as well as environmental contamination. Moreover, mass spectrometers used to analyze exhaled breath are very expensive. According to our expert in exhaled breath analysis, we do not recommend using an offline mass spectrometer; the available results have been obtained with online devices that immediately analyze samples and provide m/z values for VOCs, which are more accurate and precise [43].

Hence, there remains a lack of clear understanding of the pathophysiological pathway(s) associated with the release of these VOCs in the exhaled breath of IHD patients. To clearly determine the origin of VOCs in the exhaled breath of IHD patients, strict inclusion and exclusion criteria are required (excluding patients with various diseases known to affect the components of exhaled breath, as in this study, NCT06181799), along with a definitive diagnosis of IHD using validated tests, including a visualization test in accordance with clinical cardiology guidelines. A large cohort is essential for validating the results and enabling clinical application. In addition, building a decision tree analysis using machine learning may help to demonstrate the full potential of the molecular pathways, from biochemical reactions within the organism to detection by mass spectrometry.

## 9. Conclusions 

The lack of standardized guidelines for using exhaled VOCs for diagnosis, prognosis, early detection, and patient follow-up during treatment has led to highly heterogeneous VOC measurement results, including during oxidative stress. Therefore, the current focus is on developing a single guideline for the use of various exhaled breath methods, biostatistical analysis, machine learning models for analyzing large volumes of data, and devices for analyzing exhaled breath.

To use exhaled breath analysis in clinical practice, further research is required, including larger cohort studies and external validation of the built machine learning models.

## Abbreviations

VOCs, volatile organic compounds; IHD, ischemic heart disease; ROS, reactive oxygen species; RNS, reactive nitrogen species.
